# IL-1 family cytokines trigger sterile inflammatory disease

**DOI:** 10.3389/fimmu.2012.00315

**Published:** 2012-10-09

**Authors:** John R. Lukens, Jordan M. Gross, Thirumala-Devi Kanneganti

**Affiliations:** Department of Immunology, St. Jude Children’s Research HospitalMemphis, TN, USA

**Keywords:** Sterile inflammation, IL-1, IL-18, IL-33, NLRP3, inflammasome, caspase-1, autoinflammatory disease

## Abstract

Inflammation plays vital roles in protective responses against pathogens and tissue repair, however, improper resolution of inflammatory networks is centrally involved in the pathogenesis of many acute and chronic diseases. Extensive advances have been made in recent years to define the inflammatory processes that are required for pathogen clearance, however, in comparison, less is known about the regulation of inflammation in sterile settings. Over the past decade non-communicable chronic diseases that are potentiated by sterile inflammation have replaced infectious diseases as the major threat to global human health. Thus, improved understanding of the sterile inflammatory process has emerged as one of the most important areas of biomedical investigation during our time. In this review we highlight the central role that interleukin-1 family cytokines play in sterile inflammatory diseases.

## INTRODUCTION

Infection and cellular injury are the two principal stimuli that provoke inflammation. Detection of infectious agents and danger signals by pattern recognition receptors orchestrates a coordinated series of events that ensure removal of the insult and promote tissue repair. Following recognition of the foreign agents a cascade of inflammatory cytokines are induced that instruct the recruitment of neutrophils and macrophages to the site of infection or injury. Once mobilized to the tissue sites neutrophils and macrophages engulf and contain the insult, and also recruit additional immune cells to the site of inflammation through the production of cytokines and chemokines. Many of the pathogen-derived factors that trigger inflammation have been formally characterized, and include conserved molecules that are required for microbial survival. In comparison, sterile activators of inflammation are structurally diverse and can originate from both endogenous and exogenous sources. For instance; mechanical trauma, hypoxia, metabolic distress, chemical and environmental insults, and ischemia can all provoke sterile inflammation. Emerging data suggests that man-made and environmental irritants (silica, asbestos, alum, alloy particles, and car exhaust), metabolic factors (cholesterol, amyloids, saturated fatty acids, and glucose) and endogenous danger signals that are released as a result of aberrant cell death [ATP, reactive oxygen species (ROSs), uric acid, and interleukin-1α (IL-1α)] can all trigger sterile inflammation (Rock et al., 2010).

**FIGURE 1:**
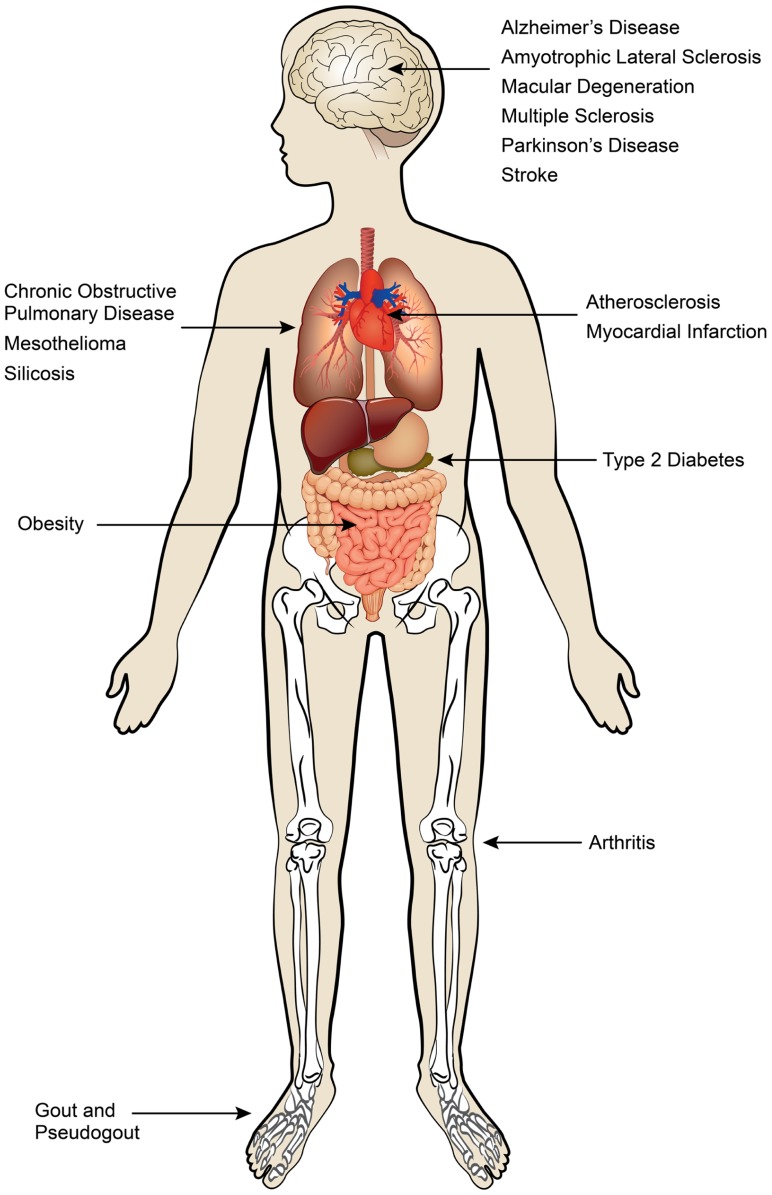
**Sterile inflammatory diseases.** Sterile inflammatory diseases can affect multiple organ systems and are a major threat to global human health.

Although inflammatory responses play critical roles in the eradication of pathogens and sterile insults, excessive and unremitting inflammation causes damage to healthy tissue and centrally contributes to disease pathology. Dysregulated production of cytokines, ROSs, proteases, and growth factors by both innate and adaptive immune cells can lead to collateral damage and disrupt tissue homeostasis. Sterile inflammation has been implicated in a spectrum of acute and chronic disorders that include obesity, atherosclerosis, type 2 diabetes, gout, and multiple neurodegenerative diseases (**Figure 1**). In this review we focus on the pivotal role of IL-1 family cytokines in various sterile inflammatory diseases.

### IL-1 FAMILY CYTOKINES

The IL-1 family of cytokines consists of 11 members that are centrally involved in regulating inflammatory responses to both infections and sterile insults. IL-1 family cytokines include IL-1α, IL-1β, IL-1Ra, IL-18, IL-33, IL-36Ra, IL-36α, IL-37, IL-36β, IL-36γ, and IL-38 (Dinarello, 2011). In this review we specifically focus on the roles of IL-1α, IL-1β, IL-18, and IL-33 in sterile inflammatory disease as these cytokines have been described to significantly influence disease pathogenesis. The emerging roles of the other IL-1 family cytokines in biology are beyond the scope of this review and are described in detail elsewhere (Dinarello, 2010).

Interleukin-1 has been shown to promote sterile inflammatory disease pathogenesis at multiple levels. For example, IL-1 can directly cause tissue destruction, altered fibroblast proliferation, and collagen deposition (Schmidt et al., 1984; Zucali et al., 1986; Ishida et al., 2006; Steer et al., 2006). Moreover, IL-1 receptor (IL-1R) signaling potently induces the production of secondary inflammatory cytokines and chemokines such as IL-6, TNFα, KC, and G-CSF (Di Paolo et al., 2009; Orjalo et al., 2009). IL-1 also contributes to the perpetuation of inflammatory disease by promoting the induction of pathogenic cytokines (IFN-γ, IL-17, and GM-CSF) by T cells and innate effector cells (Sutton et al., 2009; Lukens et al., 2012). Much like IL-1, IL-18 has also been shown to stimulate proinflammatory signaling and has historically been classified as a potent inducer of IFN-γ production. Likewise, IL-18 also promotes the activation and recruitment of inflammatory immune cells including macrophages, neutrophils, natural killer (NK) cells, and T cells (Nakanishi et al., 2001; Dinarello, 2007). IL-33 is one of the newest members of the IL-1 cytokine superfamily and its roles in inflammatory disease are just now being uncovered (Liew et al., 2010). Engagement of the ST2 receptor by IL-33 provokes the induction of T helper 2 (Th2) cytokines including IL-5 and IL-13, and thus IL-33 uniquely contributes to the pathogenesis of Th2-mediated inflammatory diseases. Below we describe the molecular mechanisms that control the release of these potent cytokines and highlight emerging data that suggest central roles for IL-1 family cytokines in sterile inflammatory diseases.

### INFLAMMASOME-DERIVED IL-1β AND IL-18

**FIGURE 2:**
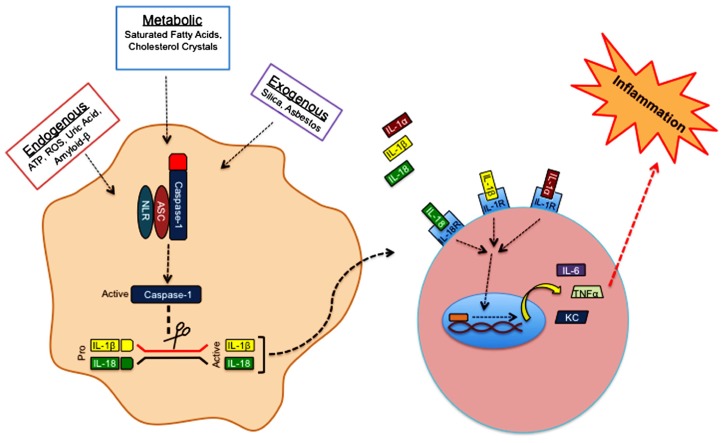
**Sterile inflammasome activation.** Endogenous, metabolic, and exogenous danger-associated signals activate NLRP3 in the cytosol. Detection of danger signals by NLRP3 promotes the recruitment of ASC and caspase-1 into a multi-protein complex known as the inflammasome. Inflammasome formation provides the necessary molecular platform to promote self-cleavage and activation of caspase-1. Activated caspase-1 cleaves the pro-forms of IL-1β and IL-18, which is required for their secretion and biological activity. Inflammasome-derived IL-1β, and IL-1α that is released from dying cells bind IL-1R, whereas IL-18 has its own receptor. Engagement of IL-1R or IL-18R promotes the robust production of secondary proinflammatory molecules (TNFα, IL-6, KC, etc.) that can then trigger additional immune cell recruitment and activation.

Over the past decade extensive research from various groups has shed light on the ability of inflammasome complexes to regulate the processing and activation of IL-1β and IL-18. Inflammasomes are comprised of a Nod-like receptor (NLR) or a pyrin- and HIN-200 domain-containing protein (PYHIN), an adaptor protein, and caspase-1 (**Figure 2**). NLRs are sensor molecules that detect both pathogen- and danger-associated molecular patterns (PAMPs and DAMPs, respectively). Activation of NLR proteins promotes the recruitment of the inflammasome-adaptor protein, ASC (also known as PYCARD), and pro-caspase-1 into a molecular platform known as the inflammasome. These multi-protein complexes mediate the proximity-induced autoactivation of caspase-1. Active caspase-1 subsequently cleaves pro-IL-1β and pro-IL-18, which is required for their secretion and to elicit their inflammatory properties. The activation of inflammasomes can occur in response to an array of different stimuli. For instance, inflammasome-mediated recognition of pathogen-derived molecules is a critical first line of defense during infection (Kanneganti et al., 2006; Kanneganti, 2010; Lupfer and Kanneganti, 2012). On the other hand, inflammasomes are also involved in the detection of danger- and stress-associated signals that are generated during sterile inflammation. Sterile stimuli that trigger inflammasome activation include endogenous danger signals that are released during aberrant cell death (ATP and uric acid), metabolic factors (saturated fatty acids and cholesterol crystals), and exogenous irritants (asbestos and silica; **Figure 2**). Mutations in NLR-inflammasome proteins are associated with both monogenic and polygenic human inflammatory disorders. Most of these rare genetic disorders are associated with mutations that result in exacerbated secretion of inflammasome-derived cytokines (Aksentijevich et al., 2007). Importantly, therapeutics that block IL-1 signaling have proven successful in the treatment of these disorders (Hoffman et al., 2004; Church and McDermott, 2009).

### INFLAMMASOME-INDEPENDENT SOURCES OF IL-1β

Bioactive IL-1β can also be generated by inflammasome-independent mechanisms, however, the contributions of these non-canonical sources of IL-1β to the inflammatory environment and disease pathology are poorly understood. Proteases that are expressed by neutrophils are primarily responsible for the majority of caspase-1 independent IL-1β. Examples of neutrophilic proteases that have been shown to cleave pro-IL-1β independently of caspase-1 activity include elastase, proteinase-3, cathepsin G, granzyme A, and chymase (Dinarello, 2011). Roles for inflammasome-independent IL-1β in disease pathogenesis have only been described in a few settings to date. For instance, capase-1 autonomous IL-1β was reported to provoke sterile inflammation in models of urate crystal-induced peritonitis and joint damage (Guma et al., 2009; Joosten et al., 2009). Cell death that ensues in sterile inflammatory environments results in the passive release of inactive pro-IL-1β. Extracellular pro-IL-1β can then be cleaved and activated by activated neutrophils that express surface-bound proteases (Netea et al., 2010). As most sterile inflammatory diseases are associated with enhanced neutrophil recruitment and tissue damage, it is likely that protease-dependent activation of IL-1β is involved in pathogenesis at some level. One possible scenario is that inflammasomes are involved in the initial sensing of the sterile threat and that caspase-1 independent sources of IL-1β are important in perpetuating the inflammatory environment later in the response. Regardless, it is clear that additional studies are needed to elucidate the unique contributions of inflammasome-independent sources of IL-1β in sterile disease.

### IL-1α RELEASE

Historically, IL-1α and IL-1β have been believed to possess overlapping biological functions. Indeed, it is true that recombinant IL-1α and IL-1β both bind to IL-1R to induce a proinflammatory signaling cascade. However, several lines of evidence point to distinct biological roles for IL-1α. For one, IL-1α belongs to a family of “dual function cytokines”, which can exert distinct biological functions in the nucleus and also when released into the extracellular compartment. The nuclear location sequence in the N-terminus of IL-1α allows it to translocate to the nucleus where it affects transcription (Buryskova et al., 2004; Werman et al., 2004). Both IL-1α and IL-1β are first synthesized as precursor proteins that can be enzymatically cleaved. The precursor form of IL-1β is not biologically active and requires cleavage to elicit its inflammatory activity. In comparison, the precursor form of IL-1α is biologically active. However, under certain poorly defined situations, precursor IL-1α can also be cleaved by the calcium-activated protease calpain to release the N-terminal propiece and produce the mature form of IL-1α. The secreted mature form of IL-1α can then bind IL-1R to induce inflammation. Why IL-1 exists in three different biologically active forms is an important question in the field. Furthermore, the discrete roles of each form of IL-1α (precursor, propiece, and mature) in disease progression remains to be formally addressed.

**FIGURE 3:**
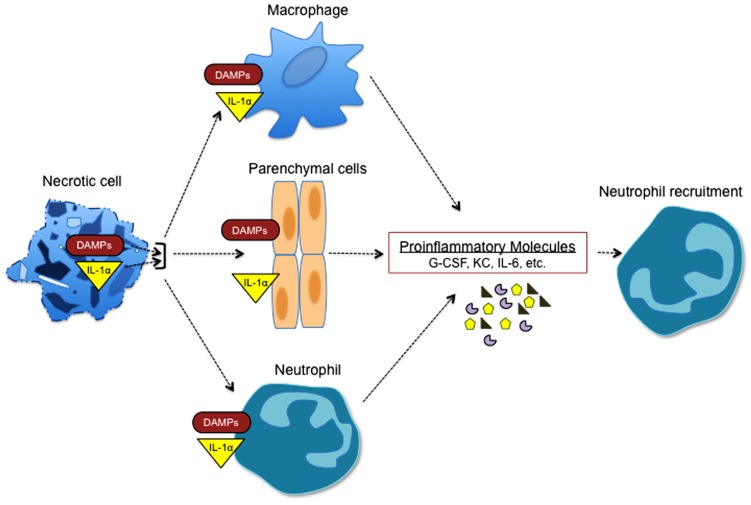
**Necrotic cells trigger sterile inflammation.** Necrotic cells potently induce sterile inflammation through multiple mechanisms. IL-1α that is released from necrotic cells induces the generation of cytokines and chemokines that are associated with granulopoiesis and neutrophil recruitment (G-CSF, KC, IL-6, etc.). In addition, multiple danger-associated molecular pattern molecules (DAMPs) including uric acid, reactive oxygen species (ROS), and ATP are released during necrosis and can promote the expression of factors that are involved in inflammation and neutrophil recruitment.

Under homeostatic conditions cells typically undergo apoptotic cell death, which does not provoke inflammation. However, cellular stress that occurs in response to trauma, hypoxia, chemical and environmental insults, and complement-mediated lysis can promote necrotic cell death. During programmed apoptosis IL-1α is trafficked to the nucleus to prevent its release into the extracellular compartment (Cohen et al., 2010). In contrast, necrosis is associated with the release of IL-1α, which then acts on macrophages, neutrophils, and parenchymal cells to trigger production of IL-6, TNFα, KC, G-CSF, and other inflammatory mediators (**Figure 3**). The passive secretion of IL-1α by necrotic cells following trauma or sterile insults orchestrates the recruitment of neutrophils and macrophages to the site of injury where they are needed to sequester the injurious agent, remove the dead cells, and initiate the healing process (Chen et al., 2007; Eigenbrod et al., 2008). However, exacerbated IL-1α release leads to pathogenic neutrophilic responses and collateral tissue damage, and thus is centrally involved in disease progression.

A recent report also suggested that IL-1α is secreted in a caspase-1 dependent fashion (Gross et al., 2012). They showed that processed IL-1α is secreted in high amounts following stimulation with various inflammasome activators. Intriguingly, caspase-1 catalytic activity was not required for IL-1α secretion in these scenarios. In the future it will be important to define the physiological relevance of catalytic independent caspase-1-mediated IL-1α release *in vivo*. Regardless, it is clear that a more complete understanding of the cellular and molecular pathways that regulate IL-1α processing and secretion is critically needed. These findings will aid in the design of novel autoinflammatory therapeutics and also provide insight into the etiology of sterile inflammatory diseases.

### IL-33

Interleukin-33 is a newly described member of the IL-1 family that is predominantly expressed by non-hematopoietic cells including endothelial and epithelial cells (Moussion et al., 2008). Similar to IL-1α, IL-33 has also been characterized as a dual function cytokine that can exert transcriptional activity in the nucleus (Carriere et al., 2007). Originally it was thought that caspase-1 is required for the processing and secretion of IL-33, however, it was later found that active IL-33 release occurs independently of capsase-1 (Talabot-Ayer et al., 2009). Paradoxically, it has been formally shown that full-length IL-33 is fully bioactive and that caspase-mediated cleavage of IL-33 actually results in its inactivation (Cayrol and Girard, 2009; Luthi et al., 2009). Release of IL-33 is believed to occur passively during cell death (Luthi et al., 2009; Talabot-Ayer et al., 2009).

Interleukin-33 signals through the ST2 receptor, which is abundantly expressed on Th2 cells, mast cells, and most other immune cell populations (Ali et al., 2007; Chackerian et al., 2007). Stimulation of Th2 cells with IL-33 induces secretion of the Th2 cytokines IL-4, IL-5, and IL-13 (Schmitz et al., 2005). In a similar fashion, IL-33 has also been shown to drive potent production of Th2 cytokines by type 2 innate lymphoid cells (ILCs; Spits and Cupedo, 2012). Furthermore, engagement of ST2 by IL-33 on mast cells has been suggested to centrally contribute to anaphylactic shock by triggering the production of proinflammatory cytokines and degranulation (Pushparaj et al., 2009).

## ROLE FOR IL-1 FAMILY CYTOKINES IN STERILE INFLAMMATORY DISEASES

### NEURODEGENERATIVE DISEASE

Interleukin-1 is a potent neurotropic cytokine that has been implicated in numerous neurodegenerative diseases including Alzheimer’s disease (AD), stroke, Parkinson’s disease, and amyotrophic lateral sclerosis (ALS; Allan et al., 2005). These diseases are typically associated with elevated local and systemic levels of IL-1. Furthermore, IL-1 can cause neuronal cell injury, breakdown of the blood–brain barrier, and astrogliosis (Shaftel et al., 2008). In the case of AD, fibrillar peptide amyloid-β (Aβ) accumulation that accompanies dementia and neuronal cell death was found to incite NLRP3 inflammasome-dependent IL-1β production (Halle et al., 2008). Genetic deletion of NLRP3/caspase-1/IL-1β axis molecules markedly attenuated the production of proinflammatory cytokines and neurotoxic factors in responses to Aβ, and also abrogated microglia activation in the brain. Genetic studies have also recently identified the *Il33* gene as a potential genetic determinant of AD (Chapuis et al., 2009). In this study they show that *Il33* expression is markedly reduced in AD patients. Intriguingly, they demonstrate that IL-33 overexpression hinders the production of Aβ. Additional studies are needed to follow-up on this exciting link and also to define how IL-33 mechanistically limits Aβ secretion.

Recent advancements in IL-1 cytokine family biology have also uncovered important roles for these proinflammatory mediators in both ALS and Parkinson’s disease. The most common genetic cause of ALS is dominant gain of function mutations in superoxide dismutase 1 (SOD1). Recently it was described that mutant SOD1 accelerates ALS pathogenesis through the induction of inflammasome-derived IL-1β (Meissner et al., 2010). In the case of Parkinson’s disease, polymorphisms in *Il1β* are associated with exacerbated neuropathology, and IL-1β was found to increase the rates of dopamine neuron degeneration in a Parkinson’s disease mouse model (McGeer et al., 2002; Koprich et al., 2008).

Ischemic or hemorrhagic conditions that are responsible for strokes result in the rapid induction of proinflammatory cytokines and inflammatory cells. The initiation of this inflammatory cascade ultimately causes neuronal cell death and functional impairment. IL-1 expression is one of the most highly up-regulated factors in stroke patients, thus randomized clinical trials using IL-1R antagonist (anakinra) to treat acute stroke were conducted (Rothwell et al., 1997; Wang et al., 1997; Emsley et al., 2005). Strikingly, stroke patients treated with anakinra exhibited reduced proinflammatory cytokine levels and improved cognitive functions.

Collectively, these findings highlight a critical role for inflammasome-induced inflammation and tissue damage in various neurodegenerative diseases. Moreover, they have helped to improve our understanding of the etiology of neurodegenerative disorders and have uncovered novel pathways to target in the treatment of these debilitating diseases.

### PULMONARY DISEASE

The lung is constantly exposed to a multitude of airborne pollutants. Deposition of environmental or man-made irritants in the lung is known to cause fibrosis and extensive cellular infiltration. Prolonged damage can ultimately result in devastating pulmonary disease. IL-1R signaling has emerged as a crucial regulator of many irritant-induced lung diseases. For instance, IL-1R deficient mice are highly resistant to lung damage in a myriad of chemically induced pulmonary disease models (Gasse et al., 2007). In these studies, disruption of IL-1 signaling was shown to attenuate proinflammatory cytokine production, immune cell recruitment, and fibrosis in response to a variety of irritants including bleomycin, cigarette smoke, diesel fuel, silica, and asbestos (Cassel et al., 2008; Hornung et al., 2008; Wilson et al., 2010; Yazdi et al., 2010). Moreover, it was clearly demonstrated that NLRP3 is a primary sensor of airborne pollutants in the lung. Indeed, asbestos and silica potently induce inflammasome activation and IL-1β secretion following NLRP3 recognition. Aerosolized asbestos-exposure in NLRP3 deficient mice resulted in diminished recruitment of inflammatory cells to the lung and concomitant reductions in proinflammatory cytokines (Dostert et al., 2008). Collectively, work in this field suggest that IL-1 blocking therapeutics may prove beneficial in the treatment of asbestos-induced mesothelioma, silicosis, and potentially other chronic obstructive pulmonary diseases (COPD).

Important roles for IL-33 in airway inflammation have also been recently described. In particular, IL-33 has been found to pivotally contribute to asthma-induced pathology. Expression of IL-33 is highly up-regulated in the lungs of asthmatic patients and in mouse models of asthma (Kurowska-Stolarska et al., 2008; Prefontaine et al., 2009). Damage to epithelial cells lining the airways is believed to be the major source of IL-33 that triggers asthmatic flares (Schmitz et al., 2005). IL-33 release promotes the recruitment of dendritic cells, Th2 cells, eosinophils, and mast cells into the airways and ultimately results in Th2-mediated inflammation (Liew et al., 2010; Borish and Steinke, 2011). Direct injection of IL-33 into the lungs of mice has been shown to rapidly induce eosinophilic inflammation and airway-hyperresponsiveness (AHR; Kondo et al., 2008; Kurowska-Stolarska et al., 2008). Furthermore, in the ovalbumin (OVA)-induced mouse model of airway inflammation, IL-33 causes exacerbated lung damage and inflammatory cell infiltration (Kurowska-Stolarska et al., 2008). Treatment of OVA-induced AHR mice with ST2 and IL-33 blocking antibodies was also found to ameliorate airway inflammation, which highlights the great promise that IL-33 neutralizing therapies hold in the treatment of asthma (Lohning et al., 1998; Coyle et al., 1999; Liu et al., 2009).

### ATHEROSCLEROSIS

Atherosclerosis is an inflammatory disorder that occurs when fats and cholesterol accumulate around the arterial walls and cause disruptions in blood flow and heart failure. Numerous lines of clinical data point to instrumental roles for IL-1 family molecules in the induction and progression of atherosclerosis. For instance, the expression of IL-1β and IL-1R are markedly up-regulated in arterial plaques, and expression levels are linked to disease severity (Moyer et al., 1991; Galea et al., 1996). Moreover, circulating IL-18 levels can be utilized to predict the risk of atherosclerosis-related death in patients (Mallat et al., 2001; Blankenberg et al., 2002). Recently, the NLRP3 inflammasome has been identified as a central regulator of atherosclerosis pathogenesis. It was shown that cholesterol crystals incite NLRP3 inflammasome activation and subsequent IL-1β and IL-18 secretion (Duewell et al., 2010). Important roles for inflammasomes have also been established in other mouse models of atherosclerosis. For example, LDL-receptor-deficient mice (genetically prone to atherosclerosis) that are reconstituted with bone marrow cells from mice that lack NLRP3, ASC, or IL-1β are remarkably resistant to plaque formation (Duewell et al., 2010). The apolipoprotein E (APOE) mouse model is also routinely utilized to study the events that are responsible for atherosclerosis pathogenesis. APOE deficient mice on a high fat diet (HFD) develop severe hypercholesterolemia and arterial plaques around the heart. Interestingly, blockade of IL-1 signaling by treatment with IL-1R antagonists or genetic ablation of IL-1R results in marked resistance to the development of atherosclerosis in the APOE model (Chi et al., 2004). These findings suggest that IL-1 contributes to HFD-induced arterial plaque formation. However, a recent study also found that genetically crossing APOE mice to mice that lack NLRP3 does not diminish the severity or incidence of atherosclerosis (Menu et al., 2011). It remains unclear why these two different mouse models of atherosclerosis have produced opposite findings in regards to the role of the NLRP3-inflammasome in cardiovascular inflammation. Therefore, additional studies are needed to formally elucidate the role of IL-1 in atherosclerosis models.

In contrast to the pathogenic role of IL-1 and IL-18 in atherosclerosis pathogenesis, IL-33 has been described to attenuate cardiovascular inflammation and plaque formation (Miller et al., 2008). Administration of IL-33 to APOE deficient mice promotes the induction of Th2-associated cytokines (IL-4, IL-5, and IL-13) that play a protective role in atherosclerotic plaque formation. Furthermore, IL-33 treatment also limits plaque development and cardiovascular disease by promoting the production of atheroprotective anti-oxidized low-density lipoprotein (oxLDL) antibodies and limiting macrophage foam cell maturation (Miller et al., 2008; McLaren et al., 2010).

### ISCHEMIA-INDUCED INFLAMMATION

Disruption in blood flow to organs causes aberrant cell death and is responsible for triggering ischemia-associated disease. Inflammation arises due to aberrant cell death and hypoxia in the organ. Myocardial infarction, hypoglycemia, hypotension, and surgery can trigger ischemia-induced inflammation and tissue damage. Altered IL-1 signaling has been identified to be a major culprit in the pathology of many ischemia-related diseases (Boutin et al., 2001; Thornton et al., 2010). Indeed, massive production of both IL-1α and IL-1β occurs during ischemia, and blockade of IL-1 signaling can significantly attenuate tissue damage and cytokine production (Shito et al., 1997; Pomerantz et al., 2001; Abbate et al., 2008; Luheshi et al., 2011). The release of the major alarmin molecule, IL-1α, following necrosis and hypoxia-induced cell death is a crucial initiating step of the ischemia-induced inflammatory cascade (Chen et al., 2007). In contrast, inflammasome-mediated IL-1β production by macrophages is believed to play important roles in sustaining local inflammation later in the response (Rider et al., 2011).

Recently emerging data suggests that IL-33 plays a protective role during ischemia-induced inflammation. IL-33 is highly expressed in both mice and humans following myocardial infarction, and studies utilizing mice that are deficient in ST2 suggest that IL-33 limits tissue destruction that results from myocardial infarction (Weinberg et al., 2002; Sanada et al., 2007; Seki et al., 2009). Soluble ST2 levels also directly correlate with impaired left ventricular function post myocardial infarction and thus circulating ST2 levels has been proposed as a biomarker for heart failure (Weinberg et al., 2003).

### JOINT AND BONE DISEASE

Chronic inflammatory bone and joint diseases can cause debilitating pain, physical impairments, and significant morbidity. Importantly, the rates of these diseases are projected to rise substantially in coming years due to increased life expectancies, sedentary lifestyles, and the ongoing obesity epidemic. IL-1 can affect various aspects of bone and joint integrity, and as a result IL-1 is critically involved in the pathogenesis of rheumatoid arthritis (RA), periodontal disease, osteoarthritis, and gout (Walsh et al., 2006; Takayanagi, 2007; Jones et al., 2011). For instance, IL-1 stimulates bone resorption bone by directly impinging on osteoclast and osteoblast functions (Nguyen et al., 1991; Kwan Tat et al., 2004). On the other hand, IL-1 signaling contributes to cartilage deterioration by impairing chondrocyte proteoglycan synthesis and stimulating the production of joint damaging mediators such as matrix metalloproteinases and nitric oxide (Joosten et al., 2004; Zwerina et al., 2007). In the case of RA, insufficient levels of the naturally occurring IL-1R antagonist in the synovium are associated with joint pathology (Firestein et al., 1994). Treatment of RA with IL-1 pathway inhibitors has achieved moderate clinical success and is currently prescribed to limit the progression of joint damage in individuals with moderate to severe RA (Arend et al., 1998; Bresnihan et al., 1998). Similar to RA, dysregulated IL-1-mediated events also impinge on the development of osteoarthritis; however, therapeutic targeting of IL-1 has only provided modest improvements in clinical trials (Chevalier et al., 2009).

Gout and pseudogout are sterile inflammatory disorders that occur when monosodium urate (MSU) or calcium pyrophosphate dihydrate (CPPD) crystals, respectively, deposit in the joints and periarticular tissue. These crystalline agents cause aggravated inflammation and neutrophil recruitment that can result in bone and joint damage. This sterile inflammatory response is dependent on NLRP3 inflammasome activation both *in vitro *and *in vivo* (Martinon et al., 2006). Moreover, colchicine, which is commonly used to treat gout and pseudogout, was found to dampen inflammasome-triggered IL-1β production. Currently, inhibition of crystal uptake is targeted to treat gout, however, this recent data suggests that addition of IL-1R blockade could substantially improve current therapeutics.

Excessive Th1- and Th17-mediated inflammatory responses have traditionally been associated with joint destruction, especially in the case of arthritis. IL-33 has been extensively shown to potently induce Th2-driven inflammation, and thus it came as a great surprise that the absence of IL-33-mediated signaling results in attenuated joint disease (Xu et al., 2008). Appreciable levels of IL-33 and ST2 can be detected in the synovium of RA patients (Carriere et al., 2007; Palmer et al., 2009). Furthermore, the disruption of ST2 activation, either by genetic abrogation or antibody-induced blockade, resulted in decreased joint disease in the mouse model of collagen-induced arthritis (CIA; Xu et al., 2008; Palmer et al., 2009). The lack of functional ST2 contributed to protection in this model through the dampening of inflammatory cytokine and anti-collagen antibody production. In contrast, treatment of CIA-induced mice with exogenous IL-33 at the onset of disease resulted in exacerbated joint destruction. Expression of ST2 on mast cells was shown to be critical for disease pathology in the CIA model, thus suggesting a critical role for IL-33-mediated activation of mast cells in joint disease (Xu et al., 2008).

### MACULAR DEGENERATION

Age-related macular degeneration (AMD) is the leading cause of vision loss and blindness in older adults. It is characterized by the accumulation of protein aggregates – known as drusen deposits – between the retina and choroid of the eye. Recently, the importance of inflammasome-derived IL-18 in this debilitating eye disease was described (Doyle et al., 2012; Tarallo et al., 2012). In one study it was shown that the accumulation of *Alu* RNA transcripts that are associated with disease progression in AMD patients can activate the NLRP3 inflammasome (Tarallo et al., 2012). Using both genetic and pharmacological inhibition of inflammasome components they found that the NLRP3/ASC/caspase-1 axis and IL-18, in particular, was required for ocular damage in response to *Alu *RNA. Intriguingly, they showed that regulation of inflammasome activation in ocular-specific epithelial cells and not immune cells was involved in disease pathology. A separate study also reported critical roles for inflammasome-derived IL-18 in AMD. Their findings revealed that drusen droplets isolated from AMD patients incite inflammasome activation and the subsequent release of IL-1β and IL-18 (Doyle et al., 2012). However, in contrast to the other study, NLRP3 inflammasome-induced IL-18 was found to have a protective role in macular degeneration. It is currently unclear why these two recent studies have yielded disparate findings, however, differences in mouse models may account for the different outcomes. Regardless, it is clear from these studies that inflammasome-induced IL-18 critically regulates ocular damage.

### MULTIPLE SCLEROSIS

Interleukin-1 signaling has also been found to play instrumental roles in the pathogenesis of multiple sclerosis (MS) in animal models and patients. Mice that are deficient in IL-1R are protected from the development of neuroinflammation and demyelinating disease in the experimental autoimmune encephalomyelitis (EAE) mouse model of MS. Disruption in IL-1R was shown to confer protection by abrogating the induction of pathogenic CD4^+^ T cells and γδ T cells (Matsuki et al., 2006; Sutton et al., 2006; Chung et al., 2009). Moreover, IL-1R^-^^/^^-^ mice exhibit marked reductions in IL-17 and GM-CSF production (Sutton et al., 2009; Lukens et al., 2012). Genetic ablation of caspase-1 provided significant protection during the early phase of the response, however, inflammasome deficiency did not lead to the same levels of protection as seen in mice that lack IL-1R (Gris et al., 2010; Shaw et al., 2010; Inoue et al., 2012a). This suggests that inflammasome-independent sources of IL-1 are also important in the pathogenesis of EAE. Future studies are needed to further characterize these sources of IL-1 in neuroinflammatory disease.

IFN-β is routinely prescribed to treat MS and the mode of action of this therapy was recently described to involve inhibition of inflammasome-mediated IL-1β production (Guarda et al., 2011a). Interestingly, one-third of MS patients do not respond to this regimen (Inoue et al., 2012b). It is possible that inflammasome-independent sources of IL-1β drive neuroinflammation in MS patients that are non-responsive to IFN-β. Strategies that inhibit inflammasome-independent IL-1 generation may hold great promise for the treatment of MS in this group of patients.

Both protective and pathogenic roles have been recently assigned to IL-33 in EAE. In one study, it was found that genetic abrogation of ST2 promotes exacerbated neuroinflammation (Jiang et al., 2012). They suggest that IL-33 provides neuroprotection by dampening IL-17 and IFN-γ production, and also by promoting the induction of alternatively activated macrophages that possess suppressive functions. Another study, on the other hand, showed that blockade of IL-33 during the induction phase can limit the development of demyelinating disease by curtailing the expression of pathogenic cytokines (Li et al., 2012). Both studies agree that IL-33 and its receptor, ST2, are highly expressed in the central nervous system (CNS) of EAE mice. Additional studies on the role of IL-33 in EAE are thus needed to clarify these disparate results.

### OBESITY AND METABOLIC SYNDROME

The incidence of obesity worldwide has increased at a staggering rate in recent decades and as a result the obesity epidemic has become a major threat to global human health. Obesity is associated with increased rates of type 2 diabetes, atherosclerosis, and joint disease. Current strategies to combat obesity that include lifestyle and dietary changes have been unsuccessful in curtailing the obesity epidemic. Thus, therapies that target the specific molecular pathways that promote obesity-related diseases are desperately needed and are at forefront of biomedical investigation. Immune cell-mediated inflammation is now recognized to contribute to the development and progression of obesity (Gregor and Hotamisligil, 2011; Osborn and Olefsky, 2012).

Extensive research has shown that IL-1 family cytokines drive the development of obesity and related diseases. Of note, obesity progression in both diet-induced and genetically prone obese mice coincided with enhanced caspase-1 and IL-1β activity (Stienstra et al., 2010; Vandanmagsar et al., 2011). Importantly, genetic and pharmacological abrogation of the inflammasome was demonstrated to lower obesity-associated inflammation and improve insulin-sensitivity in HFD fed mice (Masters et al., 2010; Stienstra et al., 2010, 2011; Vandanmagsar et al., 2011; Wen et al., 2011). During obesity the adipose tissue undergoes considerable expansion and remodeling to store excess energy in the form of fat (Shoelson et al., 2006). Differentiation of adipocytes during adipose tissue expansion is associated with the activation of caspase-1, and IL-1β has been reported to convert adipocytes into a more insulin-resistant phenotype (Stienstra et al., 2010). Furthermore, hypertrophic and hyperplastic changes that occur during weight gain are typically associated with increased cell death and the release of cell death-related stimuli (ATP, uric acid, ROS, and damage mitochondria) in metabolic tissue can trigger inflammasome activation and enhanced secretion of IL-1β and IL-18 (Strissel et al., 2007; Khan et al., 2009).

Saturated fatty acids and other obesity-related metabolites that are markedly elevated in obese individuals have long been suspected to contribute to inflammation and disease pathology. However, the mechanistic link connecting metabolic factors derived from HFDs to chronic inflammation and insulin resistance remained elusive for many years. A collection of recent studies have identified the NLRP3 inflammasome as a central sensor that detects obesity-related stress signals and that triggers IL-1β production and subsequent disease pathogenesis (Masters et al., 2010; Stienstra et al., 2010, 2011; Vandanmagsar et al., 2011; Wen et al., 2011; Henao-Mejia et al., 2012). Specifically, it was discovered that saturated fatty acids (i.e., palmitate and ceramide) elicit NLRP3 inflammasome activation and subsequent release of IL-1β and IL-18 (Vandanmagsar et al., 2011; Wen et al., 2011). It is believed that saturated fatty acids disrupt AMPK signaling, which results in defective autophagy and the accumulation of dysfunctional mitochondria and ROSs. ROS production is a well-established activator of the NLRP3 inflammasome and thus the accumulation of ROS in response to fatty acid-induced impairment of autophagy is believed to be responsible for inflammasome activation in this setting (Zhou et al., 2009; Wen et al., 2011). Collectively these findings have provided us with important insight into the immunological underpinnings of obesity and define pivotal roles for the NLRP3 inflammasome in obesity-induced sterile inflammation.

### TYPE 2 DIABETES

Type 2 diabetes mellitus (T2DM) is an obesity-related inflammatory disorder characterized by insulin resistance and uncontrolled glucose levels. It has become increasing clear that inflammasome-derived cytokines centrally regulate many of the inflammatory processes that are responsible for the impairment of metabolic function and the insulin resistance that underlies T2DM. For example, IL-1β can cause apoptosis of insulin-producing β-cells in the pancreas and also promotes insulin resistance in adipocytes (Bendtzen et al., 1986; Lagathu et al., 2006). Moreover, IL-18 is up-regulated in T2DM patients and has been linked to increased secondary renal failure and atherosclerosis (Blankenberg et al., 2002; Thorand et al., 2005). Perhaps the best example of the contribution of IL-1 family cytokines in the pathogenesis of T2DM comes from the recent clinical success of IL-1R antagonists in the treatment of this chronic disease (Larsen et al., 2007). In these clinical trials, IL-1 signaling inhibition was found to stabilize blood glucose levels and improve β-cell function.

Intriguingly, islet amyloid polypeptide (IAPP), was recently discovered to stimulate inflammasome activation and exacerbate T2DM (Masters et al., 2010). IAPP is a metabolite secreted at the same time as insulin by β-cells in the pancreas. IAPP forms amyloid deposits in the pancreas during T2DM progression (Wei et al., 2011). This formation of amyloid plaques is a salient hallmark of T2DM and has been speculated to influence disease severity. Recent findings now identify that IAPP amyloid plaques impinge on T2DM pathogenesis by activating the NLRP3 inflammasome and triggering the secretion of IL-1β.

### FUTURE PERSPECTIVES AND CONCLUSIONS

Recent discoveries have identified IL-1 family cytokines as pivotal regulators of a spectrum of sterile inflammatory diseases. The effectiveness of IL-1R blockade in the treatment of T2DM and other diseases suggest that additional therapeutics that target IL-1 family cytokines may provide effective strategies to treat other devastating inflammatory disorders. The identification of the central role of IL-1-associated cytokines in inflammatory disease progression and the discovery of the specific danger signals that trigger inflammation has provided an important foundation in our understanding of the etiology of many human diseases. Despite these recent advancements, numerous important questions remain to be addressed in order to gain a more complete understanding of the role of IL-1 family cytokines in non-communicable chronic diseases.

For example the ability of inflammasomes to influence autoinflammatory pathogenesis in cell types other than macrophages and dendritic cells has not been studied in detail. Multiple immune and organ-specific cell types express NLRs and inflammasome-associated proteins (Guarda et al., 2011b; Kufer and Sansonetti, 2011). Investigation of their contribution to disease should provide novel insight. Furthermore, up to now our understanding of the role of inflammasomes in sterile inflammatory disease has been limited to findings generated from studying the NLRP3 inflammasome. Much of this can probably be attributed to the unique role that the NLRP3 inflammasome plays in sensing multiple different stress and danger signals. However, multiple new NLR–inflammasome complexes have been discovered in recent years. Evaluation of the involvement of the AIM2, caspase-11, and caspase-8 inflammasomes in sterile inflammatory disorders is a very exciting area of future investigation (Maelfait et al., 2008; Fernandes-Alnemri et al., 2009; Hornung et al., 2009; Kayagaki et al., 2011; Gringhuis et al., 2012; Vince et al., 2012). We also still lack a complete understanding of the regulatory pathways and molecules that help to dampen excessive IL-1-mediated events. Identification of key molecules and regulatory networks that facilitate in suppressing IL-1-induced inflammation is paramount to the identification of improved therapeutics to treat sterile inflammatory diseases.

Investigation of the role of commensal bacteria in sterile inflammatory disease is another burgeoning frontier in this field. Although by definition sterile inflammation diseases are non-communicable in the infectious sense, it has become apparent recently that gut microflora can shape sterile inflammatory responses. Gut commensal bacteria are involved in calibrating the activation threshold of both adaptive and innate immune cells (Abt et al., 2012; Hooper et al., 2012). Moreover, lipopolysaccharide that translocates from the gut has been reported to directly influence both obesity and cardiovascular disease (Michelsen et al., 2004; Henao-Mejia et al., 2012).

Recent advancements in the field of IL-1 biology have greatly enhanced our understanding of the etiology of numerous autoinflammatory disorders. These studies have positioned IL-1 family cytokines as central regulators that link cellular stress that results from danger/stress signals to the induction and progression of sterile inflammatory diseases. These findings and future discoveries in the IL-1 field should provide novel strategies in the treatment of autoinflammatory diseases.

## Conflict of Interest Statement

The authors declare that the research was conducted in the absence of any commercial or financial relationships that could be construed as a potential conflict of interest
